# Metabolic Engineering of Isoflavones: An Updated Overview

**DOI:** 10.3389/fpls.2021.670103

**Published:** 2021-06-07

**Authors:** Soo In Sohn, Subramani Pandian, Young Ju Oh, Hyeon Jung Kang, Woo Suk Cho, Youn Sung Cho

**Affiliations:** ^1^Biosafety Division, Department of Agricultural Biotechnology, National Institute of Agricultural Sciences, Jeonju, South Korea; ^2^Institute for Future Environmental Ecology Co., Ltd., Jeonju, South Korea

**Keywords:** genistein, isoflavones, metabolic engineering, MYB transcription factors, phenylpropanoid pathway, soybean

## Abstract

Isoflavones are ecophysiologically active secondary metabolites derived from the phenylpropanoid pathway. They were mostly found in leguminous plants, especially in the pea family. Isoflavones play a key role in plant–environment interactions and act as phytoalexins also having an array of health benefits to the humans. According to epidemiological studies, a high intake of isoflavones-rich diets linked to a lower risk of hormone-related cancers, osteoporosis, menopausal symptoms, and cardiovascular diseases. These characteristics lead to the significant advancement in the studies on genetic and metabolic engineering of isoflavones in plants. As a result, a number of structural and regulatory genes involved in isoflavone biosynthesis in plants have been identified and characterized. Subsequently, they were engineered in various crop plants for the increased production of isoflavones. Furthermore, with the advent of high-throughput technologies, the regulation of isoflavone biosynthesis gains attention to increase or decrease the level of isoflavones in the crop plants. In the review, we begin with the role of isoflavones in plants, environment, and its benefits in human health. Besides, the main theme is to discuss the updated research progress in metabolic engineering of isoflavones in other plants species and regulation of production of isoflavones in soybeans.

## Introduction

Isoflavones are a class of flavonoids mostly available in leguminous plants where they play pivotal roles in plant–microbe interactions such as rhizobia–legume symbiosis and defense responses (Sugiyama, 2019). Isoflavones are involved in nodulation process in the leguminous plants by inducing the nodulation genes (Subramanian et al., 2006). They also act as a phytoalexins in plants, i.e., compounds produced by the plants during stress or pathogen attacks (Rípodas et al., 2013). Soybeans produce the maximum amount of isoflavones of all the leguminous crops, and they are the only significant dietary source of these groups of compounds (Kraszewska et al., 2007). Isoflavones have a similar size and chemical structure to the human estrogens that binds to both estrogen α and β receptors. Therefore, they are commonly referred to as “phytoestrogens” (Messina and Wood, 2008). Isoflavones are present in soybean as glycosylated form; however, their biological activity is from their aglycones. When soy foods are consumed, the soy isoflavones are converted to their aglycones by β-glucosidase from enteric bacteria (Tsuchihashi et al., 2008).

In recent years, scientists have been increasingly interested in isoflavones because of their potential health benefits. This can also be seen in the increased number of isoflavone containing nutritional health products in the market. Isoflavones have also been linked to cancer prevention, reduced alcohol intake, prevention of osteoporosis, and cardiovascular diseases (Dixon and Steele, 1999; Pandey et al., 2014). However, this does not mean that consuming isoflavone-rich foods is the ultimate solution to preventing diseases. In certain cases, isoflavones may be needed to consume in impossible quantities to achieve desired health benefits, although there may be some negative effects on human health. Therefore, it is imperative to increase the level of isoflavones in natural environment through metabolic engineering. Hence, understanding the molecular mechanism of isoflavone biosynthesis in various crops is important. This could pave the way to improved production of isoflavone and subsequently helps in the functional food production.

## Structure and Natural Role of Isoflavones in Plants and Environment

**FIGURE 1:**
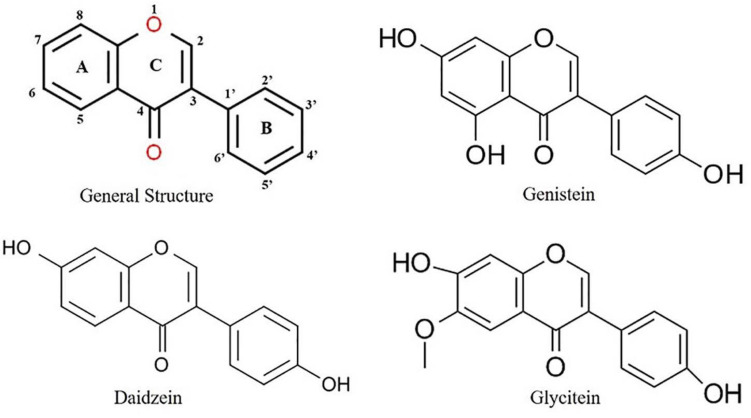
Structure of important isoflavones.

The general structure of isoflavone (Figure 1) is made up of a 3-phenylchromen-4-one backbone, with the rings denoted by the letters A, C, and B, beginning from the left (Figure 1). The position of phenyl ring in the structure of an isoflavone varies from that of a flavone, which is in position 2 in flavone but in position 3 in isoflavones (Figure 1). Although these compounds have structural similarities, they differ in their chemical behavior, and hence, the synthetic approaches for flavones cannot be used with isoflavones. Isoflavones belong to the large isoflavonoid family, which includes the following groups: isoflavones, isoflavans, isoflavanone, isoflavonols, isoflav-3-enes, α-methyldeoxybenzoins, rotenoids, pterocarpans, coumestans, 2-arylbenzofurans, 3-arylcoumarins, and coumaronochromones (Reynaud et al., 2005).

**FIGURE 2:**
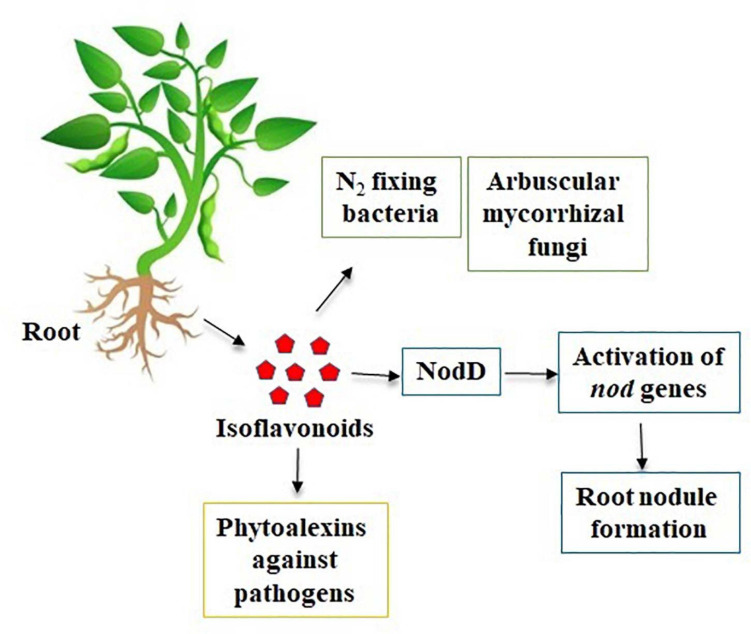
Natural role of isoflavones in plants and environmental interactions.

Isoflavones are known to have many effects on plant–microbe interactions, including control of nodulation, having an antifungal activity, and being precursors to phytoalexin (Yu et al., 2000). Phytoalexins act against both prokaryotic and eukaryotic microorganisms with their large spectrum of defense mechanisms (Paxton, 1981). In soybean, both the simple and complex derivatives of isoflavones act as phytoalexins. The main isoflavone phytoestrogens are genistein, daidzein, and glycitein (Křížová et al., 2019) (Figure 1). A rapid increase in the isoflavonoid levels in soybeans has been reported upon treatment with various pathogens (Graham, 1991, 1995; Lozovaya et al., 2004; Jeandet et al., 2013). Isoflavones also play a role in plant–environment interactions by mediating the symbiosis between plants and N_2_ fixing bacteria. As plants could not use atmospheric N_2_, the legumes develop symbiosis with the bacteria and used for its metabolism (Mulligan and Long, 1985). In respect to plant–microbe interaction, the rhizobia attracted by the root exudates move toward the legume roots through positive chemotaxis (Gaworzewska and Carlile, 1982; Caetano-Anolles et al., 1992; Compton et al., 2020). The *Rhizobium* genes are classified into two classes. Genes that determine the synthesis of exopolysaccharides (*exo* genes), lipopolysaccharides (*lps* genes), capsular polysaccharides of K antigens, and β-1,2-glucans (*ndv* genes) belong to the one class of genes involved in the synthesis of bacterial cell surface (Iyer and Rajkumar, 2017). The second class of genes comprises nodulation (*nod*) genes. Isoflavonoids from the plants act as key factor to induce the activation of rhizobial nodulation genes (Philips and Tsai, 1992; Liu and Murray, 2016; Ahmad et al., 2020) and require the participation of the transcriptional-activator protein NodD (Figure 2). In the first step, flavonoids excreted by the plant form a complex with the NodD protein, promoting the transcription of bacterial nod genes (Fisher and Long, 1992; Oldroyd et al., 2011; Del Cerro et al., 2019). In the second step, the bacterium produces lipooligosaccharide signals (Nod factors) (Spaink, 1992; Del Cerro et al., 2019) that cause various root responses through structural nod genes (Spaink et al., 1991). The role of isoflavonoids in root nodule formation is extensively studied in soybean by overexpression and RNAi-mediated gene silencing of *IFS* (*isoflavone synthase*) genes (Subramanian et al., 2005). This is not only specific to N_2_ fixing bacteria, but it also plays an important role in symbiosis with mutualistic fungi (Figure 2). Isoflavones may promote spore germination, hyphal formation and growth, root colonization, and arbuscule formation within the root during the establishment of fungal symbiosis (Abdel-Lateif et al., 2012). The specific characteristics of utilization of atmospheric N_2_ for their metabolism signify the legumes as important plant species for the development of soil quality and as an alternative for chemical N_2_ fertilizers.

## Role of Isoflavones in Human Health

**FIGURE 3:**
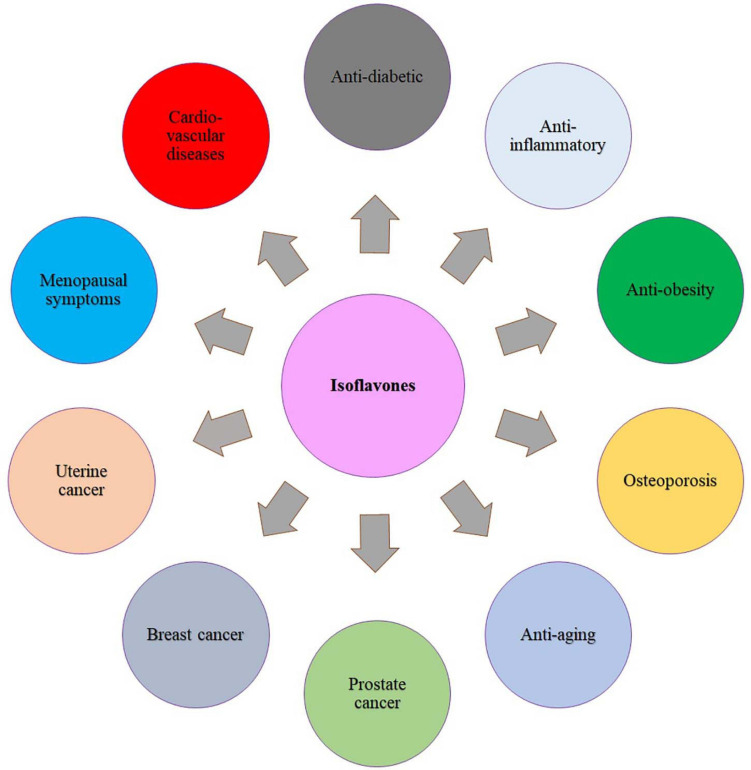
Benefits of isoflavones in human health.

In the last few decades, the consumption of isoflavone-rich functional foods is highly recommended owing to the potential health protection against some aging-associated diseases such as cardiovascular disease, osteoporosis, hormone-related cancer, and cognitive impairment (Gilbert and Liu, 2013; Vitale et al., 2013; Chi et al., 2016) (Figure 3). Even though isoflavones present in a variety of plant-derived products such as cereals, potatoes, vegetables, and fruits, the richest sources in the human diet are soy-derived foods (Kraszewska et al., 2007). For instance, the US Food and Drug Administration announced in 1999 that consuming soy protein (25 g/day) (i.e., soy isoflavone) on a daily basis may decrease the risk of coronary heart disease by reducing the blood cholesterol content. Various clinical studies have revealed that isoflavone favorably lowers the risk of cardiovascular disease because of its estrogenic property (Yan et al., 2017; Nachvak et al., 2019). A systemic review that evaluated the impact of isoflavone diet on cardiovascular disease in 1,307 menopausal (*n* = 139) and postmenopausal (*n* = 1,268) women concluded that supplementation of soy isoflavone through diet reduces the cardiovascular risk by lowering the cholesterol and triglyceride plasma concentrations and also oxidative stress (Perna et al., 2016). The clinical study by Tikkanen and Adlercreutz (2000) suggested that dietary supplementation of isoflavone reduces low-density lipoprotein cholesterol concentrations and increased high-density lipoprotein concentration. However, the recent studies demonstrated that other constituents of soy, including proteins, fiber, and phospholipids, may play an important role in balancing the cholesterol profile than isoflavones. Some evidence hints that equol, a substance converted from soy isoflavones by the action of the intestinal microflora, seems to be playing a huge role in the reduction of cholesterol content (Nestel et al., 2004; Wu et al., 2007).

As the ovarian growth hormone (estrogen) has been linked to breast and uterine cancers, isoflavones with estrogenic effects may be used as an effective treatment to thwart breast and uterine cancer (Kumar et al., 2002). However, because of the estrogen-like properties of soy isoflavones, it may stimulate the development of estrogen-sensitive breast tumors in a few cases (Messina and Loprinzi, 2001); hence, understanding the action behind the intake of soy isoflavones and breast cancer reduction is still controversial. In contrast to these conclusions, epidemiologic results suggest that higher soy intake is linked to an approximately one-third lower incidence of breast cancer in Asian women (Wu et al., 2008). Other studies found that Japanese breast cancer patients have better survival rates than Western women, even after the diagnosis (Wu et al., 1996, 2002; Yamamoto et al., 2003). The anti–prostate cancer efficacy of isoflavones was elaborately reviewed by the Mahmoud et al. (2014). Both epidemiological and clinical studies revealed that dietary supplements containing isoflavones could be an effective alternative treatment for various hormonal disorders (Chen et al., 2019; Daily et al., 2019). One of such age-related hormonal diseases is osteoporosis, a bone loss disease that mostly occurs in women who are at menopause time. At the stage of menopause, the low secretion of ovarian hormone, i.e., estrogen, causes an imbalance between resorption and formation of new bone, and subsequent bone loss occurs (Hooper et al., 2010; Taku et al., 2011). The genistein is a well-known isoflavone phytoestrogen that plays an important role in prevention of osteoporosis by acting as an estrogen receptor modulator (Figure 3). Data from epidemiological studies revealed that the risk of osteoporosis has reduced in women who consume foods with high isoflavone content (Abdi et al., 2016).

A meta-analysis showed that supplementation of isoflavones greatly increases bone mineral density and decreases the marker of bone resorption, i.e., urinary deoxypyridinoline (Wei et al., 2012; Atcharaporn et al., 2014). They also found that there are numerous factors that could significantly influence the function of isoflavone on bone resorption and formation, such as menopausal status, dose of isoflavone, and intervention duration. Moreover, the menopausal period is associated with cardiovascular diseases due to low production of estrogen. As genistein has antiaging efficacy in various estrogen-dependent aging conditions, it can be used for cosmetic preparations to improve skin tone and reduce wrinkles and skin dryness (Wang et al., 2010; Geeta et al., 2019). The estrogenic effects of isoflavones, such as genistein and daidzein, were also used to improve the quality of sleep in Japanese adults (Cui et al., 2015). Furthermore, a previous study indicated that administration of soy isoflavones (daidzein, genistein, and glycetin) (20 mg/day) synergistically improved irritable bowel disease by combination with vitamin D in female patients (Jalili et al., 2016). Owing to its importance in natural processes and in human health, a plethora of researchers started to concentrate on the improved productions of isoflavones through metabolic engineering.

## Isoflavone Biosynthesis and the Role of Biosynthetic Genes

**FIGURE 4:**
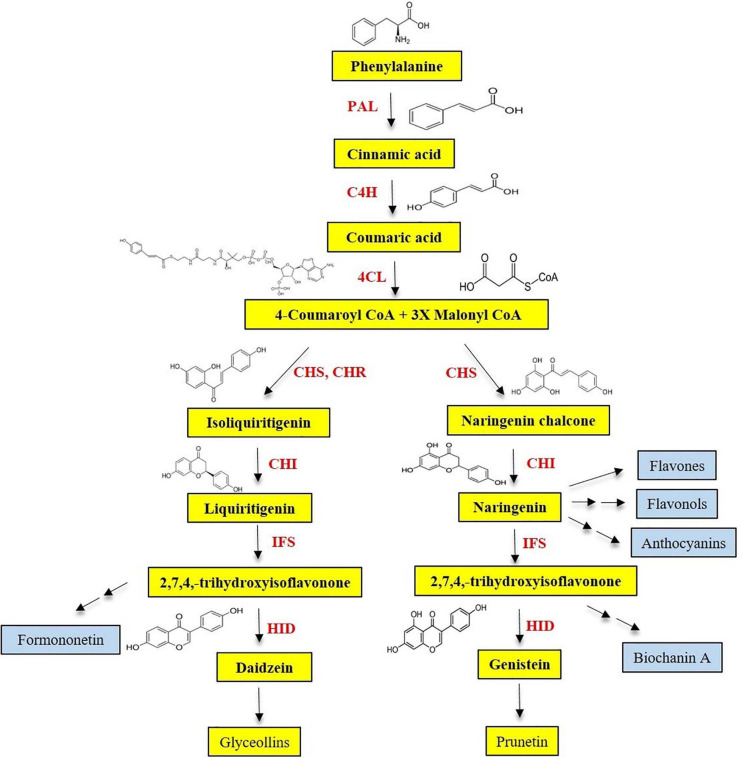
Partial phenylpropanoid pathway for the isoflavone biosynthesis.

Isoflavonoids are limited primarily to the Leguminosae and a few other species. Isoflavones are synthesized via the phenylpropanoid pathway from which plants produce most of the secondary metabolites including lignin, flavone, flavonol, anthocyanin, and tannin, etc. Isoflavones are produced by using intermediate substrates of phenylpropanoid pathway, naringenin, and liquiritigenin, respectively. Naringenin is common in most plants, and other compounds of phenylpropanoid pathway, such as flavones, flavonol, and anthocyanin, are also derived from it (Figure 4). Another intermediate substrate of phenylpropanoid pathway, liquiritigenin, is produced by chalcone isomerase (CHI) and chalcone reductase (CHR). CHI is present in most plants, whereas CHR is specific for legumes. Isoflavone reaction comprised two steps. Cytochrome P450–mediated hydroxylation associated with 2,3-aryl migration of the B ring in 2S flavanones forms a 2-hydroxyisoflavanone using IFS (Figure 4). Then, this is dehydrated into the isoflavones (genistein and daidzein) through the natural reaction catalyzed by specific dehydratase enzyme (Kochs and Grisebach, 1986; Hakamatsuka et al., 1990, 1998).

Generally, genes on the phenylpropanoid pathway are known to be triggered by environmental stresses (nutrient deficiency, excessive heat, and pathogen attack, etc.) through developmental and tissue-specific regulation (Dixon and Paiva, 1995). Many of the upstream phenylpropanoid pathway enzymes, namely, phenylalanine ammonia lyase (PAL), cinnamate 4-hydroxylase (C4H), *p*-coumaroyl-CoA (4CL), CHS, and CHI, have been well characterized. PAL catalyzes the deamination of phenylalanine to produce *trans*-cinnamic acid, which is then transformed to *p*-coumaric acid through an oxidation reaction catalyzed by C4H. After 4CL activates the thio esterification, *p*-coumaroyl-CoA directed into the branched pathway resulted in the production of lignins and flavonoids. C4H belongs to the CYP73 family of the large group of cytochrome P450 monooxygenases (Teutsch et al., 1993). Cytochrome P450 monooxygenases are playing a role in the biosynthesis of a wide range of metabolites in plants (e.g., fatty acids, phenylpropanoids, alkaloids, and terpenoids) and detoxification of herbicides and pesticides (Chapple, 1998). The accumulation of chalcone in plant tissue is rare. CHI catalyzes the stereospecific isomerization of chalcones into corresponding (2S)-flavanones, naringenin, and liquiritigenin (Figure 4). Even in the absence of CHI, chalcones can be spontaneously isomerized to form (2S)-flavanones, at a slower rate (Jez et al., 2000). CHR, which is not present in nonlegume species, belongs to the aldo-keto-reductase superfamily and is involved in the synthesis of 6′-deoxychalcone, which is the precursor for daidzein. Among the genes, flavanone-3-hydroxylase (F3H) uses naringenin as a substrate; therefore, it competes with IFS for the formation of isoflavones. The phenylpropanoid pathway genes and their roles are elaborated below.

### Phenylalanine Ammonia-Lyase Genes

Phenylalanine ammonia-lyase is a key enzyme in the phenylpropanoid pathway that catalyzes the first step of the pathway (Kervinen et al., 1997; Wang et al., 2007) found in plants (Ritter and Schulz, 2004; Francini et al., 2008), fungi (Hyun et al., 2011), and bacteria (Xiang and Moore, 2005). There is a positive correlation relationship between PAL activity and anthocyanin content in various fruits such as grapes (Hrazdina et al., 1984), strawberries (Given et al., 1988), and apples (Steyn et al., 2004), whereas negative correlation was detected in litchi fruit (Wang et al., 2004). *PAL* genes have been transformed and characterized in different plant species, including tobacco (Nagai et al., 1994), ginkgo (Cheng et al., 2005), Mongolian milkvetch (Liu et al., 2006), banana (Wang et al., 2007), Salvia (Hu et al., 2009), coffee (Lepelley et al., 2012), and so on.

Synthesis of phenylpropanoids is regulated by multiple steps, including the entry of sugars into the shikimic acid pathway, Phe into the general phenylpropanoid pathway, and the activated coenzyme A (CoA) esters into various subbranches of the phenylpropanoid pathway. PAL catalyzes the nonoxidative deamination of L-Phe to produce cinnamic acid, a reaction that is thought to be a central control point for which there is carbon flux into this pathway. *PAL* tends to be a gene family that exists universally in higher plants, and observation of *PAL* isoforms is common. The importance of this diversity is uncertain, but evidence of metabolic channeling within phenylpropanoid metabolism indicates that partitioning of photosynthesis into specific branches of phenylpropanoid metabolism could be involved by labile multienzyme complexes containing specific isoforms of PAL (Hrazdina and Wagner, 1985; Rasmussen and Dixon, 1999). PAL is localized in crucial metabolic position, linking the primary and secondary metabolism. PAL as a rate-limiting enzyme is thought to regulate overall flux into phenylpropanoid metabolism (Bate et al., 1994). *In vitro* PAL activity shows that feedback inhibitory regulation of its own product, *trans*-cinnamate and *trans*-cinnamic acid, was proposed to alter the transcription of *PAL* genes *in vivo* (Jorrin and Dixon, 1990; Mavandad et al., 1990; Appert et al., 1994).

### Chalcone Synthase Genes

The first flavonoid biosynthetic gene *CHS* was isolated from parsley (Kreuzaler et al., 1983; Schulze-Lefert et al., 1989). CHS forms a tetraketide intermediate that is cyclized into 4,2′,4′,6′-tetrahydroxychalcone (chalcone) using *p*-coumaroyl-CoA three malonyl-CoA extender molecules. *CHS* belongs to the multigene family, for example, 12 in petunia (Koes et al., 1989) and 8 in soybean (Matsumura et al., 2005). Few of the species has single-copy genes also such as *Arabidopsis* (Feinbaum and Ausubel, 1988) and *Petroselinum crispum* (Weisshaar et al., 1991). Alfalfa *CHS2* with three-dimensional structure reveals the active site that catalyzes chalcone formation. The catalytic center of CHS is formed by four residues (Cys164, His303, Asn336, and Phe215), which are exclusively conserved in other CHS-like enzymes, such as 2-pyrone synthase, bibenzyl synthase, stilbene synthase, acridone synthase, and the *rppA* CHS-like protein. Structural and functional studies revealed that Cys164 acts as the nucleophilic thiolate in the loading reaction and as the covalent thioester-anchor for the acyl-enzyme chain during the elongation reactions. Furthermore, in the elongation reaction, His303 and Asn336 catalyze the decarboxylation of malonyl-CoA and stabilize the intermediate state in the condensation phases of polyketide formation. Substrates and reaction intermediates at the active site are oriented by Phe215. CHS supplies the important substrate chalcone for the isoflavone biosynthesis, and its expression level plays an important role in isoflavonoid accumulation in plants (Tuteja et al., 2004). Downregulation of *CHS* genes plants may lead to the complete absence of flavones and isoflavone biosynthesis. However, these may lead to the several functions of the plants such as male sterility (Napoli et al., 1999).

### Chalcone Reductase Genes

Other than CHS, leguminous plants also have CHR, which catalyze the intermediate of the multistep CHS reaction, producing chalcone and 4,2′,4;-trihydroxychalcone (deoxychalcone) as a result of their combined catalytic activity. In response to herbivore or pathogen attacks, CHR sits at a key branch of the biosynthetic pathway and synthesizes a set of deoxychalcone-derived phytoalexins such as isoflavonoids, coumestans, pterocarpans, and isoflavans. CHR occurrence was first discovered in *Glycyrrhiza echinata* crude extracts (licorice) (Ayabe et al., 1988). Subsequently, CHR was purified, and the CHR cDNA (*GmCHR1*) from *Glycine max* (soybean) was cloned (Welle and Grisebach, 1988). Other homologs of *GmCHR* (*GmCHR2*, *GmCHR3*, *GmCHR4*, and *GmCHR2A*) have been identified and tested for their significance (Subramanian et al., 2006; Sepiol et al., 2017). Graham et al. (2007) have identified four *GmCHR* genes through mining of soybean ESTs. Until now, CHR-like enzymes have been discovered in a wide range of leguminous and some nonleguminous plant species, such as *Medicago sativa* (alfalfa) (Ballance and Dixon, 1995), *Glycyrrhiza glabra* (licorice), and *Fragaria ananassa* (strawberry) (Manning, 1998), *Pueraria lobata* (Kudzu vine), *G. echinata* (licorice) (Akashi et al., 1999), *Lotus japonicas* (Shimada et al., 2006), *Pueraria montana* (He et al., 2011), and *G. glabra* (Hayashi et al., 2013). As a result of sequence analysis, CHR was found unrelated to known ketoreductase domains of fatty acid synthases (Price et al., 2001; Cohen-Gonsaud et al., 2002) and β-polyketide synthases (Staunton and Weissman, 2001). However, aldo-ketoreductase superfamily enzymes from primary metabolism, such as aldose reductase and 3α-hydroxy steroid dehydrogenase, were found to have sequence similarities with CHR (Mameda et al., 2018).

### Chalcone Isomerase Genes

Most of the plants do not accumulate the chalcones. During the early stages of flavonoids biosynthesis, CHI catalyzes the intramolecular cyclization of chalcone and 6′-deoxychalcone both generated by the upstream enzyme chalcone synthase, into (2S)-naringenin and (2S)-5-deoxyflavanone, respectively (Holton and Cornish, 1995; Jez and Noel, 2000). CHI ensures the formation of biologically active (2S)-flavanones as chalcones naturally cyclize in solution to yield an enantiomeric mixture of flavanones. For instance, the metabolic precursor of anthocyanin pigments such as (2S)-naringenin and mutations in the CHI genes have been linked to variations in floral pigmentation (Burdick, 1958). Recently, the introduction of the petunia CHI gene was recently introduced into the tomato plants, resulting in fruits with higher flavanol content (Kang et al., 2014).

In the legume *L. japonicas*, two types of CHIs coexist with distinctive phylogenic lineages (Shimada et al., 2003). The type I CHIs are commonly present in the plant kingdom, which converts naringenin chalcone to naringenin. On the other hand, the type II CHI tends to be legume-specific and have additional catalytic activity, allowing them to convert 4,2′,4-tryhydroxychalcone (isoliquiritigenin) into (2S)-7,4′-dihydroxyflavanone (liquiritigenin). A type II CHI isolated from alfalfa (*M. sativa*) has been extensively studied structurally and mechanistically (Jez et al., 2000, 2002; Hur et al., 2004; Sun et al., 2019). According to structure–function analyses, the formation of a hydrogen bond network between the active site of CHI and its substrates appears to be important for the enzyme’s catalytic activity (Jez et al., 2002; Hur et al., 2004). Ralston et al. (2005) reported that there are four types of CHI based on the phylogenetic relationships, but types III and IV do not have chalcone cyclization activity like types I and II. Until now, CHI genes have been transformed and identified in several plants including *Pterolophia hybrida* (Van et al., 1988), *M. sativa* (Jez et al., 2000), *L. japonicas* (Shimada et al., 2003), *Oryza sativa* (Druka et al., 2003), *G. max* (Ralston et al., 2005), *Ginkgo biloba* (Cheng et al., 2011), *Ipomoea batatas* (Guo et al., 2015), tomato (Muir et al., 2001; Lim and Li, 2016), and *Chamaemelum nobile* (Wang et al., 2018), etc.

### Isoflavone Synthases Gene

Isoflavone synthases belong to the cytochrome P450 family, and they are extremely labile in the cells. In the phenylpropanoid pathway, IFS plays an important role that redirects the intermediates of flavonoid pathway to the isoflavonoid pathway. It catalyzes the committed step of isoflavonoid biosynthesis by producing the 2-hydroxyisoflavone from the flavone intermediates such as naringenin and liquiritigenin (Liu et al., 2002). The resulted 2-hydroxyisoflavone is dehydrated by the isoflavone dehydratase (HID) to produce basic isoflavone compounds such as genistein and daidzein. The IFS gene has been cloned and characterized in various plants, including *Arabidopsis*, tobacco, rice, and tomato, etc. (Table 1). Cloning of IFS encoding genes to various plant species paved way for the genetic engineering for the synthesis of isoflavone compounds in the plants which naturally do not produce isoflavones (Table 1).

**TABLE 1:** Metabolic engineering of isoflavone biosynthetic genes in various plants.

S. no.	Plants	Genes	Promoter	Purpose	References
1	*Arabidopsis thaliana*	*GmIFS*	*CaMV 35S*	Accumulation of genistein	Jung et al., 2000
2	Tobacco (*Nicotiana tabacum*) Maize BMS cell lines	*GmIFS*	*CaMV 35S*	Not identified	Yu et al., 2000
3	Maize BMS cell lines	*GmIFS and Maize CRC*	*CaMV 35S*	Accumulation of genistein	Yu et al., 2000
4	*Arabidopsis thaliana*	*GmIFS*, *MtCHI*	*CaMV 35S*	Accumulation of genistein	Liu et al., 2002
5	Alfalfa (*Medicago sativa*)	*MtIFS1*	*CaMV 35S*	Accumulation of genistein, glycosides of biochanin A, formononetin, and daidzein	Deavours and Dixon, 2005
6	Rice (*Oryza sativa* L.)	*GmIFS*	*CaMV 35S*	Accumulation of genistein	Sreevidya et al., 2006
7	Rice (*O. sativa* L.)	*maize C1-R-S*	*prolamin*	Dihydroquercetin, dihydroisorhametin, 3′-*O*-methyl quercetin	Shin et al., 2006
8	Tobacco (*N. tabacum*)	*IFS/CHI* fusion protein	*1. CaMV 35S*	Increased accumulation of genistein and genistein glycosides	Tian and Dixon, 2006
9	Tobacco (*N. tabacum*)	*GmIFS*	*CaMV 35S*	Accumulation of genistein	Liu et al., 2007
10	Lettuce (*Lactuca sativa*)	*GmIFS*	*CaMV 35S*	Accumulation of genistein	Liu et al., 2007
11	Petunia (*Petunia hybrida Vilm*.)	*GmIFS*	*CaMV 35S*	Accumulation of genistein	Liu et al., 2007
12	Tomato (*Solanum lycopersicum*)	*GmIFS2*	*CaMV 35S*	Accumulation of genistein	Shih et al., 2008
13	Tobacco	*PcIFS*	*CaMV 35S*	Accumulation of genistein and daidzein	Misra et al., 2010
14	Rapeseed (*Brassica napus*)	*GmIFS2*	*CaMV 35S*	Accumulation of Genistein	Li et al., 2011
15	Rice (*O. sativa* L.)	*PAL*, *CHS*, and *GmIFS1*	*Glu-B1* and *oleosin*	Accumulation of naringenin, kaempferol, genistein and apigenin	Ogo et al., 2013
16	Rice (*O. sativa* L.)	*Maize Lc*	*Glutelin Gt1*	Not identified	Song et al., 2013
17	Tobacco (*N. tabacum*)	*AtMYB12* and *GmIFS1*	*CaMV 35S*	Enhanced biosynthesis of isoflavones and flavonols	Pandey et al., 2014
18	Rice (*O. sativa* L.)	*SpdIFS1* and *SpdIFS2*	*Glb-1*	Accumulation of genistein	Sohn et al., 2014
19	Barrel medic (*Medicago truncatula*)	*GmIFS1*, *GmCHS7*, and *GmCHI1A*	*CaMV 35S*	Increased accumulation of genistein, daidzein, biochanin A, and formononetin	Gou et al., 2016
20	Onion (*Allium cepa* L.)	*GmIFS*	*CaMV 35S*	Increased accumulation of genistein	Malla et al., 2021

Initially, it is thought that IFS is specific to legumes and encoding genes only in leguminous plants, but researchers found that *IFS* genes in other plants such as *Beta vulgaris* (Jung et al., 2000), *Psoralea corilyfolia* (Misra et al., 2010) suggested that *IFS* can be found in other crops and medicinal plants. There are two isoflavone synthases, *IFS1* and *IFS2*, isolated from soybean which is a having high level of genetic similarity, but they have different efficiency in converting the flavones in to the isoflavones (Jung et al., 2000). Expression analysis revealed that both the *IFS1* and *IFS2* had different level of expression in different environmental conditions with *IFS1* mainly found in root and seed coat, whereas *IFS2* can be found in embryos and seed pods (Dhaubhadel et al., 2003; Subramanian et al., 2004). *In vitro* system using yeast, when the naringenin and liquiritigen were used as substrates, *IFS1* had a twofold higher activity compared to *IFS2*. During the soybean embryo development, the expression of *IFS2* gene was significantly increased for 70 days after the pollination (Dhaubhadel et al., 2007).

### Isoflavone Dehydratase Genes

Isoflavone dehydratase belongs to the carboxylesterase gene family, which is involved in the final step of isoflavonoid biosynthesis that produces the genistein and daidzein from the isoflavone skeleton. HID was first identified and purified from the *P. lobata* by rapid enzyme assay method (Hakamatsuka et al., 1998), but the amino acid sequence is not available. Compared to other genes involved in the phenylpropanoid pathway, such as *CHS*, *CHI*, and *IFS*, this gene (*HID*) is less characterized. The problem in characterizing the HID is the instability of its substrate 2-hydroxyisoflavanones. Later *HID* genes were identified and characterized from two plants, including licorice and soybean (Akashi et al., 2005). Site-specific mutagenesis in the *GmHID* revealed that the oxyanion and catalytic triad are important for the dehydratase and esterase activity of these genes. Shimamura et al. (2007) have overexpressed both *IFS* and *HID* genes in lotus, to understand the functional role of the *HID* in the isoflavone biosynthesis. The *GmHID* introduced was produced and increased the amount of genistein and daidzein in the lotus as the *GmHID* has broad specificity to both the 4′-methoxy and 4′-hydroxy substrates. The HID enzyme activity is an important limiting factor in the isoflavone biosynthesis and its level in the legumes (Du et al., 2010).

## Regulation of Isoflavone Production Through Genetic Engineering in Soybean

As the consumption of isoflavones is associated with a variety of health benefits to the humans, several attempts were made to develop soybeans that accumulate much higher levels of isoflavones than in wild-type seed. Generally, the isoflavone contents in *G. max* (soybean) were improved through metabolic engineering of the complex phenylpropanoid biosynthetic pathway. In a first study, the phenylpropanoid pathway genes were activated by expressing the maize *CRC* fusion gene, resulting in a decrease in genistein and the increase in daidzein levels with a marginal increase in total isoflavone levels (Grotewold et al., 1998). Cosuppression of flavanone 3-hydroxylase to block the anthocyanin branch of the pathway, in conjunction with *CRC* expression, resulted in higher levels of isoflavones (Yu et al., 2003). The use of transcription factor–driven gene activation combined with the suppression of a competing pathway resulted in increased isoflavone accumulation in soybean seeds. These high isoflavone soybeans could be used to make soy foods with greater health benefits to consumers.

The regulation of isoflavone biosynthesis is carried out by multiple genes and complex metabolic pathways. In order to understand the specific functions/regulation of the genes involved in the phenylpropanoid pathway for isoflavone biosynthesis, the overexpression or gene-silencing methods of specific genes can be carried out. Hence, Subramanian et al. (2005) have studied the RNAi-mediated gene silencing of *GmIFS* genes in the soybean. The study resulted in the reduced level of isoflavone accumulation in the gene silenced plants compared to control (Table 2). In another study, overexpression of *CHS6* and *IFS2* genes resulted in the reduced level of isoflavones and increased level of phenolic compounds (Lozovaya et al., 2007). Jiang et al. (2010; 2014) explored the negative regulation of F3H and *GmFNSII* genes by the RNAi-mediated gene silencing studies. Previously, the gene silencing of *GmFNSII* resulted in the increased accumulation of genistein in soybean hairy roots (Jiang et al., 2010). Later, RNAi-mediated silencing of both genes (*F3H* and *GmFNSII*) was found to increase accumulation of isoflavones such as daidzein and genistein (Jiang et al., 2014) (Table 2).

**TABLE 2:** Studies on the regulation of isoflavone biosynthesis in soybean.

S. no.	Genes	Regulation	Results	References
1	*GmIFS*	Positive regulation	RNAi-mediated gene silencing reduced the level of isoflavone content in the soybean	Subramanian et al., 2005
2	*CHS6* and *IFS2*	Negative regulation	Transformed plants found reduced accumulation of isoflavones and increased accumulation of phenolic compounds	Lozovaya et al., 2007
3	*GmMYB176*	Positive regulation	No significant improvement in the overexpression but in transient expression	Yi et al., 2010
4	*GmFNSII*	Negative regulation	RNAi-mediated silencing increased the genistein level significantly	Jiang et al., 2010
5	*GmMYB39*	Negative regulation	Overexpression reduced the transcript levels of *PAL*, *C4H*, *CHI*, and *CHS* genes	Liu et al., 2013
6	*F3H* and *GmFNSII*	Negative regulation	RNAi-mediated silencing increased the daidzein and genistein level significantly	Jiang et al., 2014
7	*GmMYB100*	Negative regulation	Overexpression reduced the transcript level, whereas RNAi-mediated gene silencing increased the transcript level of isoflavonoids genes	Yan et al., 2015
8	*GmMYB29*	Positive regulation	Overexpression increased the isoflavone level in 1.6- to 3.3-fold, whereas RNAi gene silencing decreased the isoflavone content in twofold	Chu et al., 2017
9	*GmIMaT1* and *GmIMaT3*	Positive regulation	Overexpression significantly increased the level of isoflavone aglycones, glucosides. and malonylates, whereas knockdown the genes reduced the contents	Ahmad et al., 2017
10	*GmMYBJ3*	Positive regulation	Activates CHS8 and CHI1A and increases the accumulation of isoflavones	Zhao et al., 2017
11	*Gma-miRNA12*, *Gma-miRNA24*, *Gma-miRNA26*, *Gma-miRNA28*, and *Gma-miRNA29*	Positive regulation	Corresponding genes for the *Gma-miRNA26*, *Gma-miRNA28* have increased the isoflavone content in soybean	Gupta et al., 2017
12	*GmCHI1A*	Positive regulation	Overexpression of *CHI1A* leads to the increased accumulation of daidzein in transgenic plants	Zhou et al., 2018
13	*GmMYB133*	Positive regulation	Increased the transcript level of *GmIFS2* and *GmCHS8* also increased the total isoflavone contents in the hairy roots	Bian et al., 2018
14	*GmMYB102*, *GmMYB280*, and *GmMYB502*	Positive regulation	Two- to fourfold increased accumulation of isoflavones was found	Sarkar et al., 2019
15	*GmMYB176*	Positive regulation	Activating *CHS8* gene and identified 25 metabolic genes and six metabolites upon overexpression	Anguraj Vadivel et al., 2019
16	*GmF3H1*, *GmF3H2, and GmFNSII-1*	Positive regulation	CRISPR/Cas9-mediated targeted gene editing leads to the increased accumulations of isoflavones	Zhang et al., 2020
17	*GmMYB29A2*	Positive regulation	Overexpression increased glyceollin accumulation and RNAi gene silencing decreased the accumulation. Accumulation of glyceollin leads to the resistance to *Phytophthora sojae*	Jahan et al., 2020
18	*GmCHI1A*	Positive regulation	T_2_ transgenic plants accumulated the higher level of genistein and daidzein than the control plants	Nguyen et al., 2020
19	*GmMYB176*	Positive regulation	Improved the accumulation of isoflavone biosynthetic genes based on the diurnal regulation system	Matsuda et al., 2020
20	*GmMYB176* and *GmbZIP5*	Positive regulation	Enhance accumulation of multiple isoflavonoid phytoalexins, namely, glyceollin, isowighteone, and *O*-methyl hydroxyl isoflavone in soybean hairy roots	Vadivel et al., 2021

Overexpression of *GmIMaT1*, *GmIMaT3* genes significantly increased the different forms of isoflavones such as aglycones, glucosides, and malonylates, but in knockdown of the genes, the isoflavone levels were reduced drastically (Ahmad et al., 2017) (Table 2). Zhang et al. (2020) have used a new methodology for the regulation of isoflavone biosynthetic genes by introducing CRISPR/Cas9-mediated targeting mutation of multiple genes (*GmF3H1*, *GmF3H2*, and *GmFNSII-1*) in soybean. The T3 generation of triple gene mutants produced increased levels (twice the amount) of genistein compared to controls. The increased isoflavone content also leads to the enhanced resistance to soybean mosaic virus infections (Zhang et al., 2020). Recently, Nguyen et al. (2020) found that overexpression of *GmCHI1A* was found to increase higher levels of both genistein and daidzein in T2 generations, but previously, Zhou et al. (2018) reported that it can increase the daidzein content alone (Table 2).

Not only changing the specific genes in the phenylpropanoid pathway can notably increase/decrease isoflavone content but also the transcription factors. Therefore, the identification and application of transcription factors, particularly for the isoflavone pathway, may significantly resolve this problem (Yu and McGonigle, 2005; Chu et al., 2017). The transcription factors of the Myeloblastosis (MYB) family play crucial roles in the regulation of isoflavone biosynthesis. Yi et al. (2010) demonstrated through the functional genomic approach that *GmMYB176* (R1 MYB protein) regulates *CHS8* expression and affects the synthesis of isoflavonoids in soybean. In this study, cotransfection analysis with *Arabidopsis* leaf protoplast resulted that *GmMYB176* transactivate the *CHS8* promoter with maximum activity. As a result of transient expression in soybean embryo protoplast, after 48 h, the endogenous transcript levels were increased up to 149-fold. Subcellular localization assay indicates that *GmMYB176* is a nuclear protein. RNAi-mediated gene silencing of *GmMYB176* in hairy roots resulted in reduced levels of isoflavonoids, but overexpression of *GmMYB176* did not significantly increase the levels of *CHS8* transcript (Yi et al., 2010) (Table 2). Liu et al. (2013) reported that soybean MYB transcription factor *GmMYB39* was potentially regulating the isoflavone biosynthesis. *GmMYB39* that contained N-terminal R2R3 repeats corresponds to DNA-binding domains of plant MYB-type proteins, which were highly conserved among R2R3-MYB proteins. Quantitative reverse transcriptase–polymerase chain reaction results revealed that overexpression of *GmMYB39* was found to be varied in different parts of the plants. Interestingly, the higher level of transcripts was found in flowers, and lower level in the pods. *GmMYB39* overexpression in hairy roots resulted in drastic reduction of the transcript levels of *PAL*, *C4H*, *CHS*, *4CL*, and *CHR*. However, the transcript level of IFS was slightly increased, whereas there is no change in CHI expression between overexpressed hairy roots and control roots. Overall, this report suggests that *GmMYB39* is involved in the inhibition role in regulation of isoflavone biosynthesis in soybean (Table 2).

Yan et al. (2015) found that R2R3-MYB transcription factor *GmMYB100* is involved in soybean isoflavone biosynthesis. Generally, *GmMYB100* is expressed in flowers, leaves, and immature embryo, but its level will decrease after pod ripening. The subcellular localization study found the nuclear localization of *GmMYB100*. Initially, yeast functional assay revealed the transactivation ability of *GmMYB100*, but the bioinformatics analyses suggested its negative role in flavonoid biosynthesis. Finally, the overexpression of *GmMYB100* reduced transcript levels of transgenic hairy roots and *Arabidopsis* and reduced flavonoid and flavonol productions, respectively, whereas the RNAi-mediated silencing resulted in the higher level of transcripts of six flavonoid related genes and accumulated higher level of flavonoids in transgenic hairy roots (Table 2). Similarly, Genome-Wide Association Study for the identification of SNPs related to isoflavone concentration in soybean found that another R2R3-MYB transcription factor *GmMYB29* is significantly involved in regulation of isoflavone biosynthesis (Chu et al., 2017). The subcellular localization analysis found that *GmMYB29* was located in the nucleus. It is found to be activated *IFS2* and *CHS8* gene promoters by the transient gene expression assays. Furthermore, *GmMYB29* overexpression and RNAi-mediated silencing in soybean hairy roots resulted in 1.6- to 3.3-fold increase and isoflavone contents in twofold decrease (Chu et al., 2017). Zhao et al. (2017) explored the role of *GmMYBJ3* in regulation of the isoflavone biosynthetic pathway. This result suggests that *GmMYBJ3* can activate the CHS8 and CHI1A genes; therefore, increased accumulation of isoflavones has been witnessed (Zhao et al., 2017).

Gupta et al. (2017) have identified miRNAs in the regulation of isoflavone biosynthesis in two contrast genotypes of soybean. *In silico* analysis identified 31 new miRNAs along with the 245 putative target genes from the seed-specific ESTs. Based on the Kyoto Encyclopedia of Genes and Genomes pathway analyses, five genes (*Gma-miRNA12*, *Gma-miRNA24*, *Gma-miRNA26*, *Gma-miRNA28*, and *Gma-miRNA29*) were found to be involved in isoflavone biosynthesis, among which *Gma-miRNA26* and *Gma-miRNA28* and their corresponding genes (*Glyma.10G197900* and *Glyma.09G127200*) exhibited their direct relationship with the isoflavone content of the soybean (Table 2). Bian et al. (2018) identified that *GmMYB133* (CCA1-like MYB) positively regulates isoflavone biosynthesis in soybean. Overexpression of *GmMYB133* has led to the expression of two important isoflavonoid biosynthetic genes such as *GmIFS2* and *GmCHS8* and increased total isoflavonoid contents in the hairy roots. Furthermore, the protein–protein interaction results revealed that it can form heterodimers with another isoflavone regulator *GmMYB176* and homodimers with another *GmMYB133* (Bian et al., 2018).

Sarkar et al. (2019) found that *GmMYB102*, *GmMYB280*, and *GmMYB502* were the potential transcription factors that can activate the promoters of the CHS gene (*GmCHS8*) and the IFS genes (*GmIFS1* and *GmIFS2*) in the isoflavone biosynthetic pathway by hairy root transformation assay. They have assessed the functional regulatory role of these genes by hairy root transformation assay, resulting in increased accumulation of isoflavones (two- to fourfold) in the three MYB overexpressing lines compared to vector control (Table 2). Anguraj Vadivel et al. (2019) have identified that *GmMYB176* (R1 MYB transcription factor) activates the CHS8 gene and regulates the isoflavonoid biosynthesis in soybean. They have identified 25 metabolic genes and six metabolites by the targeting approach that is differentially regulated during overexpression and silencing of *GmMYB176* in soybean hairy roots (Table 2).

Jahan et al. (2020) have identified that *GmMYB29A2* transcription factor positively regulates glyceollin biosynthesis in soybean. Glyceollins are pathogen-inducible defensive metabolites (phytoalexins) that play important roles in pathogen defense (Jahan et al., 2019). Overexpression of *GmMYB29A2* increased the expression of *GmNAC42*-1, *GmMYB29A1*, and glyceollin biosynthesis genes and metabolites, whereas RNAi-mediated gene silencing had opposite effects. Previously, Jahan et al. (2019) have studied the role of *GmNAC42*-1 in activation of glyceollin biosynthesis by expression analysis. In this study, they confirmed the positive regulation of *GmMYB29A2* that leads to the increased conversion of isoflavonoids into the glyceollin and thus develops resistance against *Phytophthora sojae* (Jahan et al., 2020). Matsuda et al. (2020) studied the diurnal metabolic regulation of isoflavones and soy saponins in soybean roots. The transcriptome and metabolite analysis of soybean plants at 6-h intervals for 48 h in a 12-h light–12-h dark condition. In the root tissues, isoflavone and soy saponin biosynthetic genes showed opposite patterns; the former are highly expressed in the day, whereas the latter are strongly triggered at night. *GmMYB176*, which encodes an isoflavone biosynthesis transcription factor, was upregulated from ZT0 (6:00 A.M.) to ZT6 (12:00 A.M.), accompanied by the stimulation of isoflavone biosynthetic genes at ZT6 (Matsuda et al., 2020). Recently, Vadivel et al. (2021) have found that the RNAi silencing of transcription factor *GmbZIP5* reduced the isoflavone accumulation in hairy roots. Furthermore, the co-overexpression of *GmMYB176* and *GmbZIP5* enhanced the accumulation of multiple isoflavonoids such as glyceollin, malonyl glycitin, isowighteone, and *O*-methyl hydroxyl isoflavone in soybean hairy roots. An ample of studies in the regulation of isoflavone biosynthetic genes provided the role of key players in soybean isoflavone biosynthesis, which could be useful for the development of soybean with desired level of isoflavones.

## Metabolic Engineering of Isoflavone in Nonsoybean Crops

Epidemiologic studies show that a high intake of soybean-derived foods is linked to a low incidence of hormone-related cancers, menopausal symptoms, osteoporosis, menopausal symptoms, and cardiovascular diseases. Furthermore, metabolic engineering of isoflavonoids in common nonlegume vegetables, grains, and fruits to increase dietary intake of these compounds has piqued researchers’ interest (Dixon and Sumner, 2003; McCue and Shetty, 2004; Deavours and Dixon, 2005).

Initially, in a monocot cell system, introduced expression of a transcription factor controlling anthocyanin pathway genes was successful in the production of genistein in the presence of the IFS gene (Yu et al., 2000). The genistein produced in tobacco, *Arabidopsis*, and maize cells is present in conjugated forms, indicating that endogenous enzymes were capable of recognizing genistein as a substrate. Introducing foreign IFS gene in *Arabidopsis* seedlings (Jung et al., 2000), tobacco petals, and maize cells in which the phenylpropanoid pathway was activated by C1 and R transcription factors resulted in successful accumulation of genistein (Yu et al., 2000), demonstrating that heterogeneous IFS can use flavanone intermediates as substrates. However, in these cases, the genistein levels were two- to threefold lesser than in soybean seeds (Dixon and Ferreira, 2002). The higher genistein content in transgenic tobacco petals compared to leaves was thought to be due to a more active phenylpropanoid pathway leading to anthocyanin biosynthesis in petals, which also increased the level of intermediates available to IFS (Yu et al., 2000). The low transgene expression is not the cause of low genistein production in the tobacco leaves. The IFS mRNA was actually detected at a higher level in the leaves than in the flowers of tobacco transformants, and IFS protein and enzyme activity were also confirmed in the leaves. Thus, the poor genistein synthesis is most likely due to a lack of naringenin substrate. As IFS can fight for naringenin in tobacco flowers and *Arabidopsis* leaves, channeling might be the cause of unavailability.

Conversely, Liu et al. (2002) produced transgenic *Arabidopsis* with significantly increased genistein accumulation (31–169 nmol/g Fresh weight (FW)), but soybean IFS was introduced into *Arabidopsis tt6/tt3* mutant in which the expression of F3H and another flavonol/anthocyanin enzyme dihydroflavonol reductase was decreased (Liu et al., 2002). As F3H and IFS both use naringenin as a substrate, the researchers hypothesized that the competition for intermediate availability between IFS and other enzymes was a limiting factor for a genistein biosynthesis in genetically modified plants. Furthermore, blocking a competing branch pathway may be a useful method to promote genistein biosynthesis (Liu et al., 2007). UV-B irradiation of *Arabidopsis* transformants expressing the soybean IFS gene resulted in a 2.5-fold increase in genistein accumulation. Increased levels of UV absorption and anthocyanins in IFS-transformed plants depict the higher activity of phenylpropanoid pathway (Yu et al., 2000; Liu et al., 2002). Deavours and Dixon (2005) have improved the genistein production for up to 50 nmol/g FW by constitutively expressing *MtIFS1* in the alfalfa plants. Genistein levels of this study are much higher than previous studies (Jung et al., 2000; Yu et al., 2000; Liu et al., 2002). Even though *MtIFS1* expresses all the tissues in plants, genistein accumulation was specific to the leaves. Apart from the genistein, the plants also accumulated other isoflavones such as daidzein and formononetin in response to the UV-B treatments.

Tian and Dixon (2006) have improved the isoflavone metabolism by introducing the CHI, CHS, and CHI/CHS fusion protein into the tobacco plants. High-performance liquid chromatography (HPLC) analysis results showed the accumulation of genistein in both IFS- and IFS/CHI-transformed plants, but not in vector control and CHI plants. In transgenic tobacco, lettuce, and petunia, both overexpression and antisense suppression were used to control the expression of multiple genes essential enzymes in the flavonoids/isoflavonoids pathway (Liu et al., 2007) (Table 1). The introduction of soybean IFS (*GmIFS*) into these plants, which lack this leguminous enzyme and therefore do not produce isoflavonoids naturally, resulted in genistein biosynthesis in tobacco petals, petunia leaves and petals, and leaves of lettuce. In tobacco, when antisense suppression of *F3H* and overexpression of *GmIFS* were done simultaneously, the yield of genistein increased prominently. In addition, overexpression of PAL also led to an enhanced genistein production in tobacco petals and lettuce leaves in the presence of IFS than in the plants that overexpressed only IFS (Liu et al., 2007). Similarly, Misra et al. (2010) transformed the IFS gene from the medicinal plant *P. corilyfolia* (*PcIFS*) into the tobacco. Overexpression of *PcIFS* resulted in the higher accumulation of isoflavones in the tobacco petals (Table 1).

As rice is an important crop and staple food for half of the world population, recently it caught extensive attention as bioreactors for the production of therapeutic compounds and proteins. Although production of isoflavones in rice plants was performed by several researchers, Shin et al. (2006) for the first time in rice plants have transformed *maize C1* and *R-S* regulatory genes for the production of isoflavones. The expression of transgenes is restricted to endosperm under the control of a rice prolamin gene promoter. The transformed plants showed several phenotypic variations such as changes in pericarp color, chalky endosperm, and opaque kernel. HPLC analysis showed that several types of isoflavones accumulated in the transgenic kernels. Among which taxifolin, 3′-*O*-methyl taxifolin and 3′-*O*-methyl quercetin were identified through liquid chromatography (LC)–tandem mass spectrometry (MS/MS) analysis (Table 1). Furthermore, they have confirmed the accumulation of isoflavones in the layers of endosperm by the florescence labeling. At the same time, Sreevidya et al. (2006) have introduced the *GmIFS* gene into the rice plants for the production of isoflavones under the control of *CaMV35S* promoter. The genistein accumulated as a glycosylated form in the rice plants. Rhizobia study for the nod gene expression confirms the accumulation of isoflavones inducing the nod genes in varied degrees.

Introduction of five biosynthetic genes (*OsPAL*, *OsC4H*, *Os4CL*, *OsCHS*, and *OsCHI*) with the endosperm-specific *GluB*-1 promoter into one vector has been done through *Agrobacterium*-mediated transformation (Ogo et al., 2013). As multiple-expression cassette is quite large, some of the parts were deleted in a few plants; as a result, none of the transgenic plants did not express all the five genes. Generally, plants were found to have one to four expression cassettes. The resulted plant that is lacking either *OsPAL* or *OsCHS* gene does not accumulate naringenin, but has both genes accumulating naringenin in the seeds. Furthermore, these selected genes were transformed exclusively with embryo- and endosperm-specific 18 kDa *oleosin* and *GluB-1* promoters for the identification of isoflavone localization. LC-MS analysis revealed the accumulation of naringenin and some flavones. Also the expression of additional genes for flavone (*PoFNSI*/*GmFNSII*), isoflavone (*GmIFS*), and flavonol (*AtF3H*/*AtFLS*) has resulted in the accumulation of kaempferol, genistein derivatives, quercetin, chrysoeriol, and tricin, etc. (Table 1). Song et al. (2013) aimed to generate colored rice seeds and increased accumulation of isoflavone that expressed *maize-Lc* gene under the control of endosperm-specific rice *glutelin Gt-1* promoter. As a result, the transgenic rice was found to be with dark color and the total isoflavone content also increased. Expression of two soybean isoflavone synthases (*SpdIFS1* and *SpdIFS2*) under the control of endosperm-specific 26-kDa *globulin* promoter in rice varieties accumulated a higher level of genistein in seeds (Sohn et al., 2014). As the two rice varieties has different seed color (black and normal white), the accumulation of isoflavone was also found to be varied. The white variety (103 μg/g) of transgenic rice has accumulated more genistein than black variety (87 μg/g) (Table 1).

In an attempt of isoflavone biosynthesis in tomato, Shih et al. (2008) have transformed soybean *IFS* (*GmIFS*) gene under the control of the *CaMV35S* promoter. The transgenic plants accumulated genistein in a tissue-specific manner. The LC-MS analysis has found that substantial amount of genistein has been accumulated in the leaves, whereas the little amount has been accumulated in fruit peel. Although *Brassica napus*, a nonleguminous oil crop, produced phenylpropanoids and flavonoids but not isoflavones because of the absence of the *IFS* gene. Incorporation of exogenous *GmIFS2* showed the accumulation of genistein in the leaves of transgenic plants up to 0.72 mg/g dry weight (Li et al., 2011) (Table 1). With the aim of increased accumulation of genistein in transgenic tobacco, Pandey et al. (2014) has engineered the tobacco with the coexpression of *GmIFS* and transcription factor *AtMYB12*. The transgenic plants accumulated higher levels of flavonols and genistein conjugates compared to control plants. Gou et al. (2016) have transformed different combinations of isoflavone pathway genes (*CHS*, *CHI*, *IFS*, and *F3H*) in *Medicago truncatula* for the increased accumulation of isoflavones and proanthocyanidins. Downregulation of *MtF3H* in combination with overexpression of *GmIFS1*, *GmCHS7*, and *GmCHI1A* was found to be more effective in elevated accumulation of isoflavones, flavones, and proanthocyanidins. Recently, Malla et al. (2021) have transformed *GmIFS* into onion (*Allium cepa* L.) for the accumulation of genistein in transformed onion plants through both biolistic gene transfer and *Agrobacterium*-mediated gene transfer methods. The results showed that a higher level of genistein was accumulated in biolistic gene transfer (62.65 nM/g FW) than *Agrobacterium*-mediated gene transfer method. Thus, introducing the transcription factor for the positive or negative regulation of the isoflavone biosynthesis proved the efficiency of transcription factors, suggesting that it can be utilized for both leguminous and nonleguminous plants for the regulation of isoflavone biosynthesis.

## Concluding Remarks and Future Prospects

Isoflavones have important roles in plants, environment, humans, and other animals with their compatible chemical structures. Their general occurrence in the soybean and its applications in various diseases, as we highlighted, necessitated the detailed understanding of the metabolic engineering process and the regulation of isoflavone biosynthesis in the plants. We have provided an overview of the isoflavone biosynthesis through metabolic engineering in various crop plants. The recent progression in the identification and characterization of important enzymes in the isoflavone biosynthetic pathway has helped to improve the level of isoflavones in plants. However, as phenylpropanoid pathway is a complex pathway for isoflavone biosynthesis, simultaneous engineering of multiple genes and understanding the crosstalk between pathways are important. That helps us to understand the role of a specific branch of the pathway in the biosynthesis of isoflavones and other secondary metabolites. The isoflavone-level variation is a complex mechanism regulated by various genetic and environmental factors. Transcriptional regulation of isoflavone biosynthesis occurs by modifying the transcription factors such as MYBs either to increase or decrease the level of isoflavones in soybean (Yi et al., 2010; Yan et al., 2015; Bian et al., 2018; Jahan et al., 2020). In addition to that, posttranslational regulation of isoflavone biosynthesis through ubiquitination and SUMOylation processes has become necessary tools for clear understanding of how isoflavones can be produced. Likewise, synthetic metabolic engineering technologies, such as a multigene expression vector system (CRISPR/cas9)–based system, should be used efficiently for expression and regulation of isoflavone biosynthetic genes in a precise manner (Zhang et al., 2020). With the deep understanding of the isoflavone biosynthetic pathway, novel technologies in metabolic engineering and synthetic metabolic engineering will decipher light on the regulation of complex metabolic networks and can produce biofortified crops with required isoflavone levels to meet better human health.

## Author Contributions

SIS and SP conceived the review and wrote the manuscript and made a critical revision of the review. YJO, HJK, WSC, and YSC performed the literature search. SP and YJO prepared figures and tables. All authors contributed to the article and approved the submitted version.

## Conflict of Interest

YJO was employed by the company Institute for Future Environmental Ecology Co., Ltd., Jeonju, South Korea. The remaining authors declare that the research was conducted in the absence of any commercial or financial relationships that could be construed as a potential conflict of interest.
